# The genetic mechanisms of the influence of the light regime on the lifespan of *Drosophila melanogaster*

**DOI:** 10.3389/fgene.2012.00325

**Published:** 2013-01-25

**Authors:** O. A. Shostal, A. A. Moskalev

**Affiliations:** Radiation Ecology, Center of Ural Division of Russian Academy of Sciences, Institute of Biology of Komi ScienceRussia

Light is a crucial environmental factor influencing living organisms during their whole lives. It contributes to the regulation of circadian rhythms, affects growth, metabolic rate, locomotor activity and reproduction. The mechanisms of the influence of light on longevity are poorly understood.

**Figure 1:**
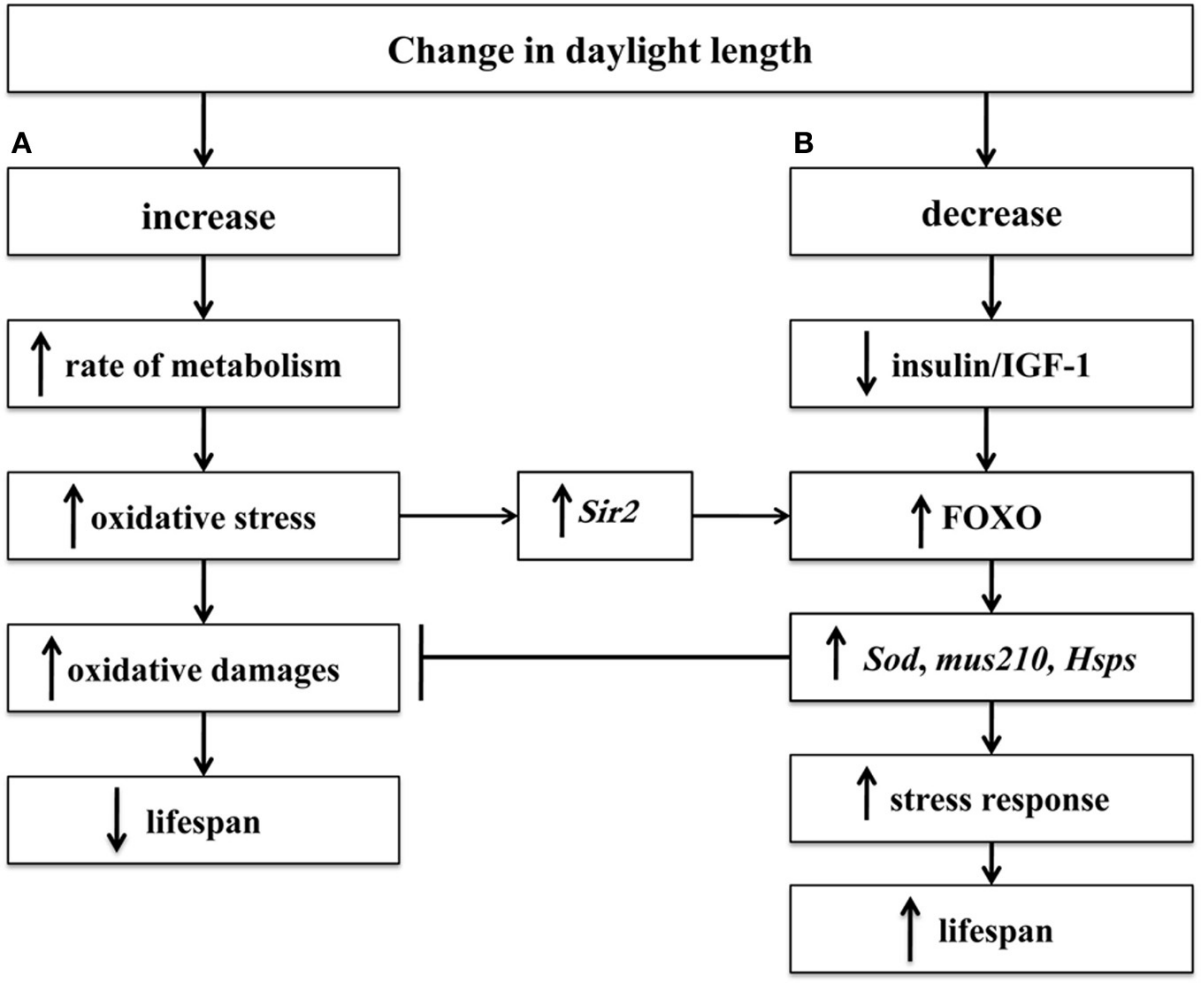
**The mechanism of the influence of the light regime on lifespan: metabolism intensification in light conditions (A) and FOXO-dependent increase of lifespan in dark conditions (B)**. Symbol: →, induction; ⊣, inhibition; ↑, increase; ↓, decrease.

We have suggested that there are two relatively independent genetic mechanisms of the influence of light on lifespan (Moskalev and Malysheva, 2010). The first mechanism is related to the damaging effects of light, leading to a reduced lifespan (Figure 1A), the second mechanism is related to the influence of the dark as a mild stressor, which can stimulate the body's defense system and lead to an increased lifespan (Figure 1B).

It is known that an increase in the photoperiod usually decreases the lifespan of experimental animals (Massie and Whitney, 1991; Massie et al., 1993; Sheeba et al., 2000; Majercak, 2002; Anisimov et al., 2004; Vinogradova et al., 2009). An increase in day length promotes a higher level of metabolism due to the intensification of locomotor activity and changes in body temperature of *Drosophila* (Sheeba et al., 2000, 2002). An increase in metabolic rate, in turn, leads to the additional formation of toxic by-products—free radicals (Massie and Whitney, 1991; Helfand and Rogina, 2003), damaging the cell's mitochondrial and nuclear DNA, membranes and proteins (Le Bourg, 2001), and as a result this can lead to accelerated aging and a reduced lifespan.

In our works (Moskalev et al., 2006, 2008) we investigated the strain *Drosophila melanogaster* with the defective cytoplasmic superoxide dismutase gene (*Sod*) which has only 36.7% of the normal activity of the *Cu/Zn Sod* enzyme (Phillips et al., 1995) and the strain with the defective *mutagen-sensitive 210* gene (*mus210*), protein-coding, involved in nucleotide-excision repair (homolog of the XPC protein in mammals) (Isaenko et al., 1994). This gene group contributes directly to the elimination of oxidative damage—through free radical detoxication (gene *Sod*) and DNA repair (gene *mus210*). It has been shown that mutations in genes responsible for the removal of oxidative damage can alter the lifespan of animal models. In particular, in *Drosophila* with zero Cu/Zn-superoxide dismutase activity, the lifespan is reduced by 80% (Staveley et al., 1990), and injecting into the short-lived strain *Drosophila* genome additional superoxide dismutase and catalase genes, basic antiradical protection enzymes, resulted in a lifespan increase (Orr and Sohal, 1994, 2003). *Sod* overexpression in *Drosophila's* motor neurons only prolonged the lifespan by 40% and made *Drosophila* more resistant to agents stimulating active oxygen formation such as ionizing radiation and paraquat (Mattson et al., 2002). We also know that the ability to repair oxidized DNA bases decreases in XPC-deficient cells (D'Errico et al., 2006). In our experiments, the median lifespan of flies with an impaired *Sod* gene function also decreased compared with the lifespan of *Canton-S* wild-type flies by 41% for males and 38% for females (Moskalev et al., 2006). In the strain with the defective *mus210* gene the median lifespan was reduced by 48% in males and 21% females compared with wild-type flies (Moskalev et al., 2006). We hypothesized that in strains with a dysfunction of *Sod* and *mus210* genes there will be a significant reduction in lifespan if subject to lighting round the clock, as compared to the wild-type strain. According to our results, in strains with *Sod* and *mus210* gene mutations, there was a significant increase in the difference between the median and maximum lifespan in the dark and in the light compared with the *Canton-S* wild-type strain (Moskalev et al., 2006). In the strain with a free radical detoxication defect, the gap in the median lifespan in the dark and in the light was 36% for males and 14% for females; the maximum lifespan was 11% for males and 24% for females. At the same time, the addition of the antioxidant melatonin into the food for *Drosophila* with this defect reduced this variation (Moskalev et al., 2008). In the strain with the DNA repair defect, the gap in the median lifespan in the dark and in the light was 11% for males and 23% for females; and the maximum lifespan was 21% for males and 9% for females. The lifespan parameters of wild-type flies varied insignificantly (within 0–7%). Thus, our results confirm our hypothesis about the importance of detoxification and DNA repair genes in the regulation of lifespan under a changing day length (Moskalev et al., 2006, 2008).

It is known that in the regulation of the oxidative stress response and lifespan a key role is played by sirtuin family proteins (Guarente and Kenyon, 2000; Balaban et al., 2005). In response to stress sirtuins deacylate histones and various transcription factors (including p53, FOXO, HSPs), activating the expression of the stress response genes and inactivating and inhibiting apoptosis, thus contributing to the cell survival rate and lifespan increase (Tanno et al., 2007; Niedernhofer and Robbins, 2008). It is known that sirtuins play a key role in the regulation of the aging rate and longevity. In particular, the ubiquitous suppression of expression of the *dSir2* and two *dSir2*-like genes (*CG5085* и *CG6284*) by means of RNA interference is lethal, and the suppression of gene expression in *Drosophila* neurons only reduces the lifespan, while at the same time, increasing the activity of sirtuins in yeast, worms and flies prolongs their lifespan (Kusama et al., 2006; Russell and Kahn, 2007; Niedernhofer and Robbins, 2008). For example, transgenic expression of *sir-2.1* prolongs the lifespan of nematodes by 50%, and overexpression of the *dsir2* gene in the nerve tissue of *Drosophila* at the larval stage prolongs the median lifespan of males and females by 20% and 52%, respectively (Rogina and Helfand, 2004). In our studies *dSir2* gene mutation led to a significant reduction in lifespan compared with the wild-type strain. The median lifespan of males and females of the strain with a deletion of the *Sir2* gene was lower than the median lifespan of the control strain by 19% in males and 33% in females (Moskalev and Malysheva, 2010). The role of sirtuins in the change of lifespan under the influence of different lighting regimes has not previously been studied. We investigated the *Drosophila* strain homozygous with deletion of the *dSir2* gene. It has been shown that the difference in the median lifespan in the dark and light was 24–33%, while in the control wild-type strain the difference in lifespan was 3–17%. Thus, our data point to the important role of the *Sir2* gene in the regulation of lifespan under a changing day length (Moskalev and Malysheva, 2010).

Equally important in the cellular stress response are heat shock proteins (Hsps) involved in the process of repair and proteolysis of damaged proteins (Hunt et al., 2004; Arya et al., 2007). Besides, higher Hsps activity is associated with the longer life in various model animals (Morrow et al., 2004; Kimura et al., 2006). The *Hsp70* gene expression intensifies after oxidative damage (Guo et al., 2007; Soti and Csermely, 2007), which contributes to the improvement of the redox status of cells and increased activity of antioxidative enzymes (Guo et al., 2007). Moreover, in *Drosophila* with mutations in genes encoding catalase and Cu, Zn-Sod, a decrease of the *Hsp70* induction period is observed with age, which provides evidence of *Hsp70* involvement in the response to oxidative stress (Landis et al., 2004). We have suggested that the *Hsp70* gene deletions in *Drosophila* can reduce lifespan in light conditions compared to dark conditions (Moskalev and Malysheva, 2010). Notwithstanding the fact that the median lifespan in the light and in the dark of the strains with *Hsp70* gene mutations changed slightly, under the exposure to light, the maximum lifespan dropped significantly (by 7–29%) (Moskalev and Malysheva, 2010).

Thus, the above evidence supports the fact that the lifespan control mechanism in varying daylight conditions is related to the damaging effects of the additional lighting.

Recently, we proposed a hypothesis which suggests that light affects the animal lifespan via neuroendocrine regulatory networks (Moskalev and Malysheva, 2009). According to this hypothesis, in response to the shortening of the photoperiod the activity of the insulin/IGF-1 signaling is decreased, and stress response machinery, including FOXO transcription factor is activated. FOXO plays a crucial role in maintaining the balance between growth and reproduction, on the one hand, and stress resistance and lifespan, on the other hand (Calnan and Brunet, 2008). It is known that FOXO mediates the response to oxidative and other stresses, which is often connected with a prolonged lifespan (Giannakou and Partridge, 2004; Vogt et al., 2005; Lam et al., 2006; Honda et al., 2010). It was shown that permanent overexpression of *dFOXO* in the fat body of an adult *Drosophila* reduces the mortality rate, stimulates the resistance to the free radical inducing factor—paraquat and prolongs the lifespan of flies (Giannakou and Partridge, 2004; Giannakou et al., 2007), whereas any defects in the functioning of *dFOXO* raises sensitivity to oxidative stress and decreases the lifespan (Junger et al., 2003). We have suggested that FOXO plays the key role in the increased lifespan when in the dark. In this case, the experimental reduction in the activity of this gene should eliminate the difference between the lifespan in the dark and in the light. Flies with reduced *dFOXO* gene function were obtained by mating flies of two strains containing the *dFOXO*^21^ and *dFOXO*^25^ alleles. In *dFOXO*^21^/*dFOXO*^25^ transheterozygotes the survival curves in the dark and under normal lighting conditions did not differ significantly in three out of four cases (Moskalev and Malysheva, 2010). This result points to the connection between the longevity of fruit flies in the dark and the activity of the FOXO transcription factor. Apparently, the minimum differences in survival of flies with reduced FOXO function in the dark and under normal lighting conditions remained because the induction of FOXO-dependent stress resistance mechanisms can occur not only in response to the suppression of insulin-like peptides in the dark, but also in response to oxidative stress in the light, adding to the reduction in lifespan in the light (Moskalev and Malysheva, 2010).

Thus, we obtained the first evidence of the role of FOXO transcription factor in the control of lifespan in varying light regimes. This role is expected to be determined by its involvement in the response to the mild stress effect of darkness.

Overall, the results of our research substantiate our hypothesis regarding the existence of two relatively independent regulatory pathways that respond to changes in lighting regimes (Figure 1). On the one hand, the increased photoperiod resulting in the intensified metabolism leads to the decreased lifespan of *Drosophila*. Actually, the strains with *Sod, mus210*, and *dSir2* gene mutations demonstrate a significantly higher difference in the lifespan in the dark and in the light with regard to the wild-type strains, and for the strains with mutations in *Hsp70* gene such regularity is a trend. On the other hand, the decreased photoperiod, although it does not cause damage, stimulates the stress response and prolongs the lifespan. In the case of reduced activity of FOXO transcription factor the increase in the *Drosophila* lifespan in the dark is poorly pronounced or absent, which substantiates our hypothesis that *Drosophila* have a FOXO-dependent mechanism of increasing lifespan in the dark (Moskalev and Malysheva, 2010).

The authors were supported by the grant from the Presidium of the Russian Academy of Science No. 12-P-4-1005; and grants from the Russian Foundation for Basic Research No. 12-04-31922 and 12-04-32261.
